# Dysentery

**Published:** 1853-01

**Authors:** F. K. Bailey

**Affiliations:** Almont, Mich.


### THE NORTH-WESTERN
### MEDICAL AND SURGICAL JOURNAL.

NEW SERIES.

VOL. I. JANUARY, 1853. No. 9.

### ORIGINAL COMMUNICATIONS.

ARTICLE I.—*Dysentery*, by F. K. Bailey, M. D., Almont, Mich.

Dysentery has prevailed to considerable extent in this vicinity during the past season, and in one locality proved very fatal. The disease was ushered in, in most cases, by a chill, soon succeeded with fever. The stools were generally similar to those in common diarrhœa, for a few hours, but soon became bloody; while, in some instances, the first unnatural discharge was composed wholly of bloody mucus. Tormina and tenesmus severe; abdomen hot, very tender on pressure, and, after a short time, more or less distended. Sometimes no external change could be detected. Tongue coated, urine scanty and red, skin hot and dry, and the pulse frequent, but feeble. Nausea and vomiting at first, in some cases, but subsiding after a day or two. Dysuria was a frequent attendant, and often very severe. Two-thirds of the cases occurred among children. The fatal cases terminated in from four to seven days, and in those that recovered, the disease continued from fourteen to twenty-one days, and sometimes longer; but generally terminating by crisis, as in fevers of the same type, at which time the alvine discharges would assume a natural appearance, and all the functions become established. The region where the disease was most severe, extends about two miles on an east and west line, and did not reach more than a mile North or South from that line. In the S. W. limit of this district is a small stream, upon which there are three mill ponds, as near each other as they can well be, overflowing more than one hundred acres of land, from which none of the timber had been removed. For twelve years, fevers common in miasmatic districts, have prevailed in this neighborhood.

The treatment most generally adopted, was the common mode—Calomel in alterative doses, or blue mass, combined with Opium, and Ipecac., followed with laxatives, &c. On watching the effects of this course I came to the conclusion, that, however well adapted it might be in ordinary cases, in this it was positively injurious; as there seemed to be a general epidemic influence operating to modify the type, rendering the disease of more than ordinary malignancy.

There was a disturbance of all the functions, with a low grade of local inflammation tending to deep ulceration and gangrene.

The alterative plan might be calculated to benefit the case as far as the general derangement was concerned, still it operated badly rather than otherwise, upon the inflamed mucous membranes; and furthermore, the local inflammation would terminate in mortification, rendering the case fatal before the constitutional difficulty would be reached by general remedies. Therefore, it seemed the most rational to direct our curative efforts to obviate the local diseased action, trusting to the efforts of nature to dispose of the general trouble.

I accordingly decided to use nothing that would act as cathartic or even laxative, but endeavored to alleviate pain, and suspend the peristaltic action of the intestines. To effect this, I gave Opium in doses sufficiently large, and often repeated, to relieve pain without inducing excessive narcotic effects. I also gave a saturated solution of common salt in strong vinegar, without being diluted, in doses of a tea-spoonful (or more if it could be borne) once in fifteen minutes until the tenesmus was relieved, which would generally take place in less than two hours, and in some cases almost instantly.

If an antacid was indicated, Sup. Carb. Soda, or Carbonate of Ammonia were most useful. As a diuretic, Spts. Nitri Dulcis was given, and the dysuria was generally relieved by a decoction of Uva Ursi. Enemata of cold water, and cloths wet in water of a temperature most grateful to the patient, laid upon the abdomen, were productive of beneficial results. The diet best suited to the case was equal parts of scalded milk and water, which would also serve as drink, with the use of Slippery-elm in water.

Nausea and vomiting were relieved by sinapisms to the epigastrium. Blistering the abdomen was dispensed with, not that it would not be beneficial in itself, but because the constant irritation on the patient's frequent getting up, would overbalance its good effects.

By following the plan above-mentioned, I found the patient suffered less pain in the bowels, the tenesmus less severe, not being felt to any amount except when on the stool. He could sleep most of the time, and it is well known by all conversant with dysentery, that tenesmus when constant is the most debilitating attendant; and it will be more or less constant when kept alive by frequent catharticizing. I would remark, in reference to the salt and vinegar, that my patients would invariably call for it, when the tenesmus was about to return, so satisfactory were its effects. How it operates to relieve that distressing sensation, I will not pretend to explain. There are more curative qualities in common salt than is generally admitted; and were it a rare and costly chemical, it would undoubtedly be more popular as a remedy among the profession than it now is. Salt and vinegar in combination are well known as a topical remedy in inflammation and ulceration of the fauces, and it probably acts upon the inflamed mucous membrane of the intestines in a similar manner.

A common error in the treatment of dysentery seems to be the practice of administering laxatives to remove the vitiated secretions from the upper intestines, when the probability is, that all that does pass off as a result of their action, is produced by the irritation of the medicine itself; and, in addition to this effect, the peristaltic action of the diseased portion of the alimentary canal, is increased, when perhaps by the effect of Opium, it may have been quieted. Opium acts not only by keeping the intestines still, but also by suspending all secretions which might irritate in their passage downwards.

Prof. Tully used to say in reference to dysentery: "Give Opium until costiveness is produced," and I have often found it a valuable hint. Should costiveness be produced it will never be necessary to give a laxative, but suspend the Opium, and the bowels will act soon enough.

There were no post-mortem examinations made in any case, in this district, but judging from the mode of attack, there must have been congestion of the mucous membrane at the time of the chill. Inflammation soon followed attended with bloody stools. In some cases reaction did not come up, but death succeeded the congestive stage. In these cases there were symptoms indicating cerebral congestion. There is every reason to conclude, that miasmatic influence operated strongly in producing the disease, or modified the predisposing causes, were they traceable to any other source than that of a paludal nature. One thing is certain, that marsh miasm has a great influence in modifying every disease, and such being the case, great care is requisite to detect any change produced by such a cause.

